# Geographic Variability of Fish Carcass–Associated Microbial Communities

**DOI:** 10.1111/1758-2229.70389

**Published:** 2026-07-22

**Authors:** Yasutake Kawamoto, Jotaro Urabe

**Affiliations:** ^1^ Graduate School of Life Science Tohoku University Sendai Japan

**Keywords:** bacteria, ciliophorans, decomposition, sardine carcass, the Japanese archipelago, tidal flat

## Abstract

Animal carcasses are common in natural environments and, owing to their high energy and nutrient content, support distinct microbial communities. However, little is known about how carcass‐associated microbial communities vary among geographically distinct regions. To address this knowledge gap, we deployed sardine carcasses on surface sediments at seven tidal flats distributed along the Japanese archipelago and compared microbial communities in sediments with and without carcasses. We focused on the taxonomic composition of bacterial assemblages and their potential predators, ciliophorans. Both bacterial and ciliophoran communities associated with carcasses differed markedly among sites. However, the overall effect of carcass addition on community composition was superimposed on strong geographic and environmental patterns. These results suggest that carcass‐associated microbial communities are shaped primarily by local species pools and environmental conditions rather than by a common carcass‐driven effect across geographically distinct sites.

## Graphical abstract

When sardine carcasses (A) were deployed across seven Japanese tidal flats (B), both bacterial (D) and ciliophoran assemblages (C) varied substantially among sites. Yet, carcass‐associated shifts (blue) were overshadowed by regional variation (red), indicating that local species pools and environmental conditions—rather than carcass inputs alone—primarily determine microbial community composition.

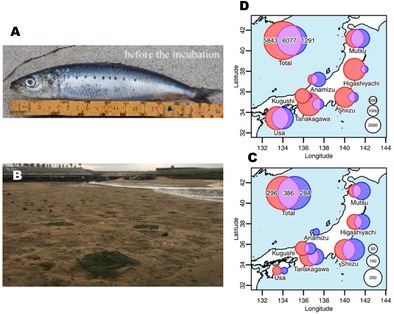

## 1 Introduction

In recent years, mass fish mortality events due to coastal stranding have been increasingly reported in the media (McCurry 2023; Baker 2024). While their causes are often debated in the context of global warming and related drivers (Genin et al. 2020; Baliarsingh et al. 2023; Tietbohl et al. 2025), the ecological consequences for surrounding coastal ecosystems remain poorly understood. Animal carcasses represent a temporary but substantial input of energy and nutrients, strongly influencing microbial communities (Parmenter and Lamarra 1991; Cederholm et al. 1999; Monaghan and Milner 2008; Beasley et al. 2012; DeBruyn et al. 2025). Such inputs typically induce pronounced shifts in microbial taxonomic composition and community structure, producing conditions that deviate sharply from baseline states (Tang et al. 2006; Goffredi et al. 2008; Premke et al. 2010; Howard et al. 2010; Pechal et al. 2013). However, most research on these dynamics has been conducted in terrestrial systems, often focusing on carcasses of large vertebrates such as pigs, humans (DeBruyn et al. 2025; Taylor et al. 2024), or wildebeest (Fouché et al. 2025).

To understand the ecological consequences of mass fish mortality in coastal ecosystems, it is necessary to examine how fish carcasses affect microbial communities and whether these effects vary across environments. To date, only one study has addressed this question: burying sardine carcasses in coastal sediments was found to substantially alter microbial community composition through bacteria–ciliophoran interactions and environmental modification (Kawamoto et al. 2021). Yet it remains unclear whether such strong effects are generalizable across geographically diverse coastal ecosystems.

The Japanese archipelago, which spans a broad latitudinal gradient and is bordered by both the Pacific Ocean and the Sea of Japan, exhibits substantial climatic and tidal variability (Naganuma 2000). Microbial communities in coastal sediments are therefore expected to vary geographically. Indeed, our previous work demonstrated spatial differences in bacterial and ciliophoran assemblages across the archipelago, driven by tidal range and sediment grain size (Kawamoto and Urabe 2023). These observations suggest that the ecological impacts of fish carcasses on microbial communities may also vary regionally.

In this study, we conducted in situ experiments at seven tidal flats across the Japanese archipelago to examine how fish carcasses affect coastal microbial communities. We focused on both bacterial and ciliophoran assemblages, given our previous finding that ciliophorans interact directly and indirectly with bacteria in shaping community structure (Kawamoto et al. 2021). To assess whether carcass effects vary geographically or with environmental conditions, we tested three hypotheses using molecular approaches (16S and 18S rRNA amplicon sequencing for bacteria and ciliophorans, respectively): (H1) the magnitude of microbial responses to fish carcasses differs among geographically different sites; (H2) microbial communities near carcasses are more similar at environmentally similar sites than at geographically proximate ones; and (H3) bacterial and ciliophoran assemblages near carcasses are interconnected through trophic interactions and environmentally mediated processes. By testing these hypotheses, we identify the ecological responses of coastal microbial communities to fish carcasses and show how these responses vary across geographical and environmental contexts.

## 2 Material and Methods

### 2.1 In Situ Incubation Experiment

Incubation experiments with fish carcasses were conducted from July to September 2019 at seven tidal flats across the Japanese archipelago (Table 1). Whole sardines ( *Sardinops melanostictus* , ~20 cm in length) were purchased from a fish market in Sendai City and frozen until use. For each experiment, six 500‐mL square plastic bottles were prepared, each perforated with 28 1‐cm holes evenly distributed on all sides. Three bottles were assigned as experimental treatments, each containing one thawed sardine carcass, while the remaining three bottles without fish served as controls.

**TABLE 1:** Summary of tidal flat locations, associated environmental conditions, and dates of the in situ incubation experiments.

Site	Location		Incubation date		Temperature (°C)		Salinity	Median particle size	TN	TOC	Ignition loss	Tidal range
	Latitude	Longitude	Start	End	Mean	Range	(psu)	(mm)	(μg/gWW)	(μg/gWW)	(%)	(cm)
Mutsu	41.1961	140.9993	2019/8/13	2019/8/20	25.5	20.4–38.6	26.27	0.1548	35.9	13.4	4.782	62
Higashiyachi	38.1895	140.9620	2019/9/5	2019/9/12	28.2	20.8–43.1	22.8	0.3141	24.6	2.23	1.229	155
Anamizu	37.2042	136.9181	2019/7/26	2019/8/2	29.4	26.5–32.1	29.33	0.281	155	18.6	2.327	39
Kugushi	35.5920	135.9032	2019/7/25	2019/8/1	28.9	22.8–48.4	1.33	0.952	37.9	3.17	1.244	37
Shiizu	35.4713	140.0303	2019/9/10	2019/9/17	26.8	18.7–49.7	15.8	0.1593	72.4	13.8	2.852	159
Tanakagawa	34.7963	136.5608	2019/9/16	2019/9/23	24.9	18.2–33.4	21.47	0.3103	39	4.35	1.108	193
Usa	33.4330	133.4385	2019/7/30	2019/8/6	29.1	26.3–38.3	11.1	0.2173	63.1	6.65	3.455	207

At each site, bottles were filled with ambient sediment, placed horizontally, and partially buried in surface sediments with one side facing the overlying water (Figure S1a). The experimental area was then covered with a mesh net securely anchored into the sediment to prevent disturbance (Figure S1b). When sardine carcasses were incubated in a tidal flat for 6 weeks, microbial community shifts were most pronounced 7 days after carcass exposure (Kawamoto et al. 2021). Based on this finding, we incubated the experimental bottles for 7 days at each site. At the end of the incubation period, sediments were collected from the bottles using a sterilized stainless‐steel core sampler (1 cm in diameter) inserted through the side holes (Figure S1c). The samples were immediately stored in a cooler (~4°C), transported to the laboratory, and then stored at −80°C until analysis.

### 2.2 Environmental Data

At each site, salinity of pore water within sediments surrounding the experimental bottles was measured at the start of the incubation experiment using a refractometer. Sediment samples were then collected from the same area to determine median particle size, ignition loss, and concentrations of total organic carbon (TOC) and total nitrogen (TN) in the pore water. Median particle size was estimated from cumulative weight curves generated by sieving dried sediment through mesh sizes of 2 mm, 1 mm, 0.5 mm, 0.25 mm, 0.125 mm, and 0.063 mm. Ignition loss was calculated as the difference in sediment weight before and after combustion at 450°C for 2 h.

For TOC and TN analyses, ~0.5 g of wet sediment was subsampled into a 15‐mL glass vial containing 10 mL of distilled water. The vial was vortexed for 10 s, centrifuged at 2000 rpm for 5 min, and an aliquot of the supernatant was transferred to a new glass vial. Samples were then diluted with 0.2‐μm filtered water to adjust concentrations to the measurable range. TOC and TN concentrations were quantified using a total organic carbon and nitrogen analyser (multi N/C 3100; Analytik Jena AG, Jena, Germany).

Sediment temperatures during incubation were continuously recorded using loggers (HOBO Onset UTBI‐001) placed on the sediment surface near the experimental bottles, and mean values over the incubation period were used in subsequent analyses. At the end of the experiment, oxidation–reduction potential (ORP) was measured in sediments collected from experimental bottles using an ORP meter.

Geographic distances between sites were estimated as sea‐route distances along coastlines using a web service (https://japonyol.net/distance.html) and are shown in Table S1. Astronomical tidal ranges at each site were obtained from the Japan Meteorological Agency (https://www.data.jma.go.jp/kaiyou/db/tide/suisan). Additional details on environmental measurements and tidal range data are provided in Kawamoto and Urabe (2023).

### 2.3 DNA Analysis

DNA was extracted from sediment samples using the PowerSoil DNA Isolation Kit (MoBio Laboratories, Carlsbad, CA). Sequencing was performed on an Illumina MiSeq platform (Illumina, San Diego, CA). For prokaryotes (primarily bacteria), the V3–V4 regions of the 16S rRNA gene were amplified using primers 342F and 806R (Mori et al. 2014). For Ciliophora, the V4 region of the 18S rRNA gene was amplified with primers TAReuk454FWD1 and TAReukREV3 (Stoeck et al. 2010). To selectively amplify Ciliophora DNA prior to 18S amplification, an initial PCR was performed using Ciliophora‐specific primers (CilF, CilR I, CilR II, and CilR III; Lara et al. 2007), and the resulting amplicons were used as templates in a nested PCR to increase specificity and sensitivity. All amplicons were sequenced in 300‐bp paired‐end mode on the MiSeq platform following the manufacturer's protocols. In this study, sediment samples exhibited high microbial sequence diversity, characterized by a large number of sequences and a high proportion of low‐abundance taxa. Under such conditions, ASVs (amplicon sequence variants), which resolve sequences at single‐nucleotide resolution, may capture even minor variations within dominant taxa, potentially complicating the interpretation of community patterns. Therefore, in this study, we employed OTUs (operational taxonomic units) as the taxonomic unit, following the procedures described in Kawamoto et al. (2021), to facilitate more robust comparisons of community structure.

For each sample, undemultiplexed sequencing data were generated using bcl2fastq (Illumina, San Diego, CA). Reads were demultiplexed by index sequences and paired‐end reads were merged using Claident (Tanabe and Toju 2013). As per Illumina's standard protocol, PhiX control sequences, added as spike‐ins, were automatically removed by the MiSeq system. OTUs were delineated at a 98% sequence similarity threshold and taxonomically annotated with the SILVAngs 1.4 amplicon analysis pipeline (Quast et al. 2013) for both bacteria and ciliophorans, as in Kawamoto et al. (2021). Chimera detection was conducted using UCHIME2 (Edgar et al. 2011) against the SILVA SSU r138.1 database for bacteria and the PR2 v4.14.0 database for ciliophorans (Guillou et al. 2013). OTU sequences were assigned to reads using VSEARCH v2.18.0 (Rognes et al. 2016). For Ciliophora analyses, non‐target OTUs (e.g., dinoflagellates and apicomplexans) were removed before downstream processing.

In some samples from Anamizu and Usa, fewer than 1000 ciliophoran reads were obtained, likely due to errors during DNA extraction or PCR amplification. To maintain sequence depth after normalization, these low‐read samples were excluded from analyses, which resulted in the removal of all control samples from Anamizu. Across all samples, the mean read depth was 19,134 reads for bacteria and 29,848 reads for Ciliophora (excluding Anamizu controls). To normalize sequencing depth across samples, rarefaction was applied to equalize read coverage (Chao and Jost 2012). OTUs frequently detected in negative controls were excluded prior to rarefaction. After quality control and normalization, the average number of reads retained for statistical analyses was 4542 per bacterial sample and 6099 per ciliophoran sample.

### 2.4 Statistical Analyses

We performed principal component analysis (PCA) on the environmental variables and used principal components (PCs) with cumulative contributions exceeding 80% of the total variance to represent environmental conditions in subsequent analyses. α‐diversity of bacterial and ciliophoran assemblages was estimated as OTU richness after rarefaction to equal read coverage across samples (see above). To evaluate the effects of environmental variables, including principal components (PCs), and fish carcass treatment (presence = 1, absence = 0), we first fitted simple regression models for each explanatory variable. Then, we constructed multiple linear models for α‐diversity with the selected environmental PCs, treatment, and their interactions as explanatory variables. A stepwise selection procedure was applied to retain only statistically significant predictors in the final models.

For each assemblage, β‐diversity was calculated using the Horn index (Horn 1966) based on proportional OTU read abundances because both OTU richness and read abundance varied substantially among sites. Variation in *β*‐diversity was visualized with non‐metric multidimensional scaling (nMDS) (Kruskal 1964). The effects of site, treatment, and their interaction on *β*‐diversity were tested using permutational multivariate analysis of variance (PERMANOVA) (McArdle and Anderson 2001). To identify factors influencing *β*‐diversity, we applied multiple regression on distance matrices (MRM) (Lichstein 2007) with five independent distance matrices: treatment score (0 if treatments were the same, 1 if different), ciliophoran *β*‐diversity, Euclidean distances of environmental PC scores, latitudinal differences, and sea‐route distances between sites. Variation partitioning (Legendre and Legendre 2012) was then conducted to quantify the independent and joint contributions of treatment and other variables to *β*‐diversity variation. The same analyses were applied to ciliophoran assemblages, with bacterial *β*‐diversity used as an explanatory variable.

Finally, we tested whether the effects of environmental PCs, sea‐route distance, and the interacting assemblage (ciliophora on bacteria and vice versa) on *β*‐diversity differed between treatments. For this, Mantel tests were performed separately for experimental and control treatments. Differences in regression slope and intercept between treatments were evaluated with a permutation‐based significance test (Legendre and Legendre 2012): (1) observed slope and intercept differences were calculated; (2) all data were pooled and permuted subsamples matching original sample sizes were drawn; (3) Mantel tests were conducted for both treatments in each of 999 permutations, and slope/intercept differences were recorded; (4) observed differences were considered significant if they fell within the top 5% of the permuted distribution. All statistical analyses were conducted in R v.3.6.3 (R Core Team 2020).

## 3 Results

### 3.1 Environmental Conditions

During the incubation period, sediment temperatures varied across sites, with the degree of variation differing among tidal flats. The highest mean temperature (29.1°C) was recorded at Usa, where values ranged from 26.3°C to 38.3°C (Table 1). In contrast, the lowest mean temperature (24.9°C) was observed at Tanakagawa, which also exhibited the widest range (18.2°C–33.4°C). Substantial variation among tidal flats was also evident in other environmental parameters, including tidal range, salinity, median particle size, ignition loss, total nitrogen (TN), and total organic carbon (TOC) (Table 1).

Oxidation–reduction potential (ORP) at the end of the experiment also varied considerably among sites in both control and experimental treatments (Figure S2). At all sites, ORP values were consistently lower in bottles containing fish carcasses than in controls. The treatment effect was particularly pronounced at Kugushi, Higashiyachi, Anamizu, and Mutsu—sites primarily located along the Sea of Japan—compared with Shiizu, Tanakagawa, and Usa on the Pacific coast.

**FIGURE 1:**
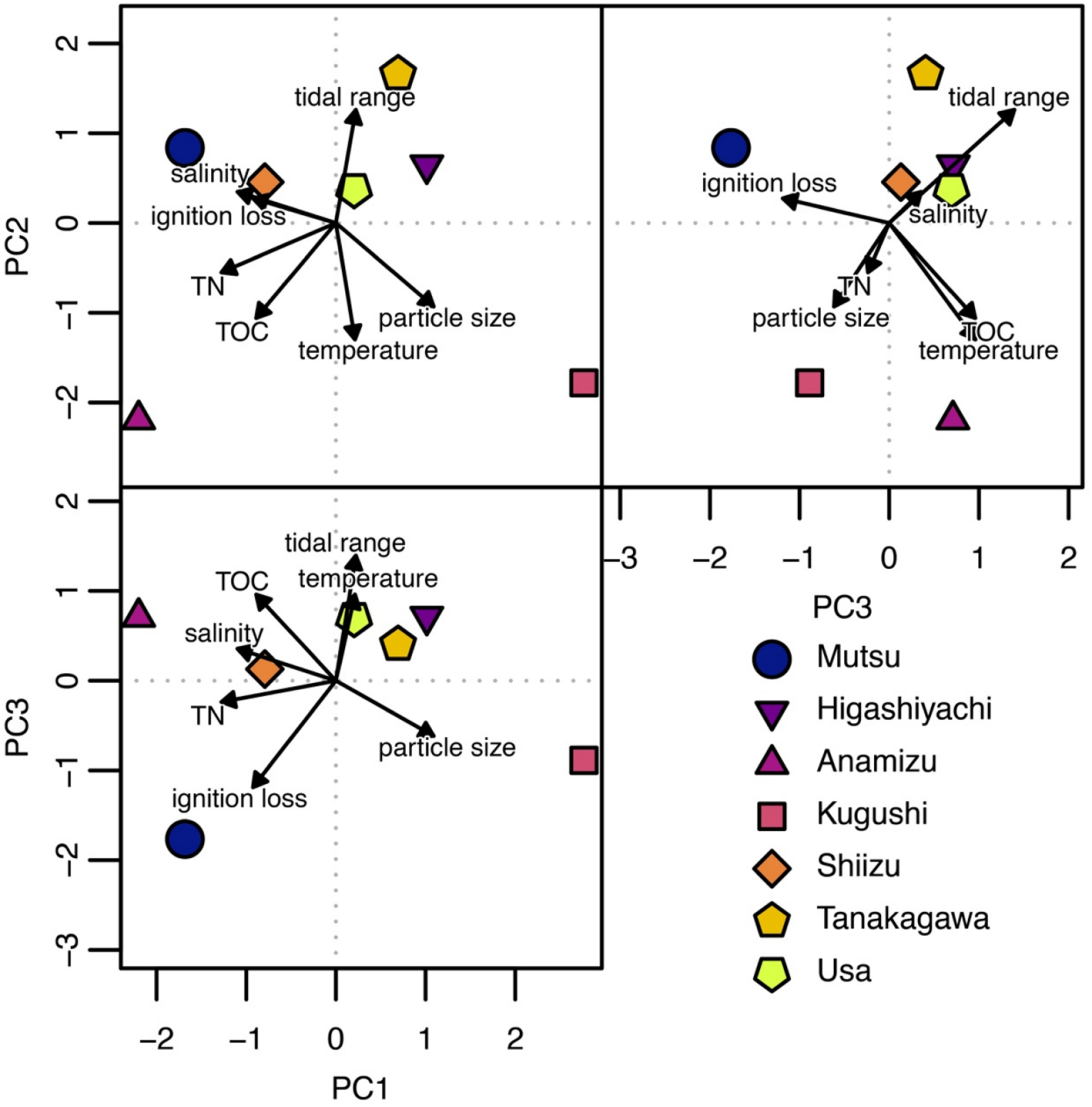
Results of a principal component analysis (PCA) based on seven environmental variables measured across seven tidal flat sites. Environmental variables are represented by arrows, and sites are distinguished by different symbols in the three‐dimensional ordination space. Statistical results of the PCA are presented in Table 2.

Principal component analysis (PCA) summarized 84% of the variation in the seven environmental variables within the first three principal components (Table 2). Based on factor loadings, PC1 represented oligotrophic conditions, with negative associations to TOC, TN, and ignition loss, and positive associations to larger particle size. PC2 described environments characterized by large tidal range and low water temperature, whereas PC3 reflected environments with large tidal range and low organic content. PCA scores highlighted marked differences among sites: although Kugushi and Anamizu are geographically close in latitude and distance, their PC1 scores diverged substantially. Moreover, Kugushi and Mutsu were clearly distinct from the other sites along PC3 (Figure 1).

**TABLE 2:** Results of principal component analysis (PCA) of environmental variables, including factor loadings, proportion of variance explained, and cumulative proportion.

	PC1	PC2	PC3
Temperature	0.088	−0.541	0.400
Salinity	−0.457	0.145	0.148
Median particle size	0.454	−0.388	−0.257
TN	−0.532	−0.229	−0.099
TOC	−0.370	−0.441	0.399
Ignition loss	−0.385	0.114	−0.495
Tidal range	0.093	0.525	0.581
Proportion of variance	0.416	0.290	0.133
Cumulative proportion	0.416	0.707	0.839

### 3.2 Bacterial and Ciliophoran Assemblages

**FIGURE 2:**
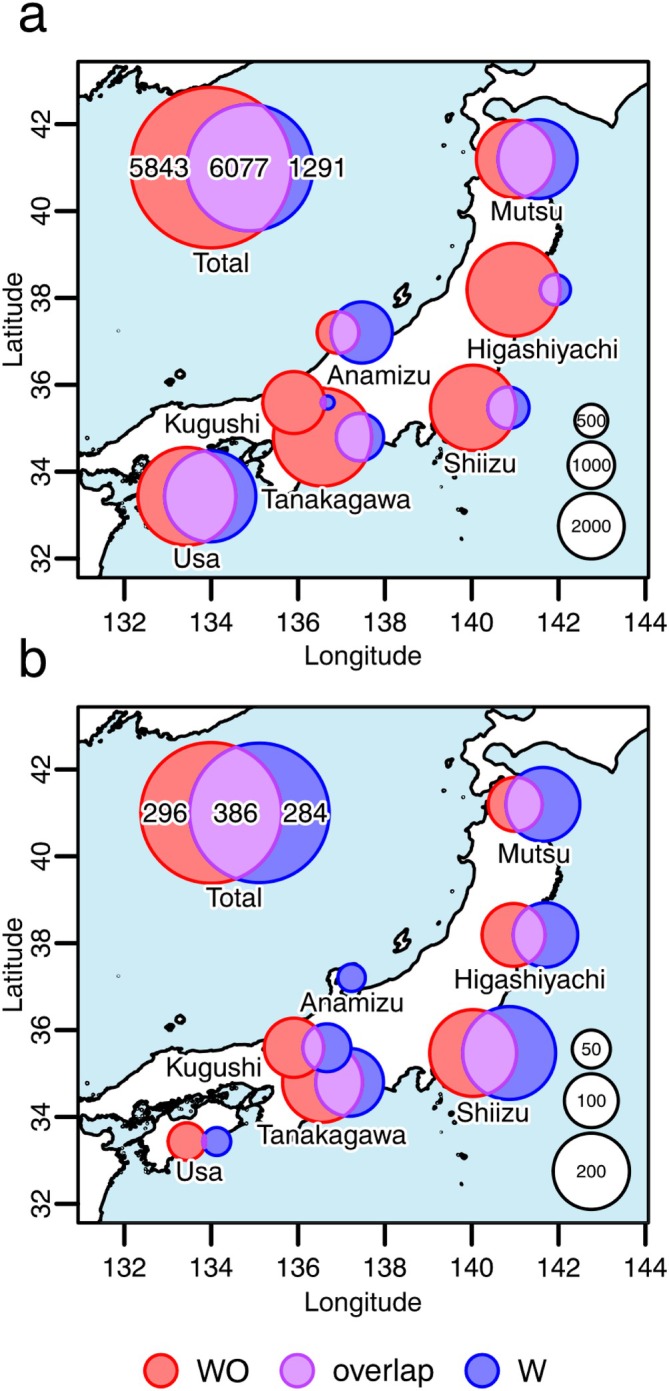
Venn diagrams showing the number of OTUs unique to control treatments without fish carcasses (WO), unique to experimental treatments with fish carcasses (W), and shared between both treatments for (a) bacterial assemblages and (b) ciliophoran assemblages at each site. The area of each region is proportional to the number of OTUs.

At the phylum level, Proteobacteria and Bacteroidota dominated bacterial assemblages across all sites (Figure S3a). Acidobacteriota and Chloroflexi were also detected but were less frequent at Higashiyachi and Kugushi. Firmicutes was particularly abundant at Kugushi, Shiizu, and Usa, whereas Fusobacteriota was present at all sites except Kugushi. In general, Firmicutes and Fusobacteriota were more frequent and abundant in experimental treatments than in controls as shown in Figure S3a. Other phyla, including Planctomycetota, Latescibacterota, Myxococcota, and Gemmatimonadota, were detected across sites but occurred less frequently in experimental than in control treatments at Higashiyachi, Shiizu, Kugushi, and Tanakagawa. At Anamizu and Kugushi, more than 70% of bacterial OTUs in the experimental treatments were absent from controls (Figure 2a). In contrast, at other sites, over 60% of OTUs in experimental treatments were also found in controls. When pooled across all sites, only 18% of bacterial OTUs were unique to experimental treatments.

For ciliophorans, classification at the class level showed Spirotrichea as the most abundant group, followed by Oligohymenophorea (Figure S3b). Spirotrichea dominated in Mutsu, whereas Oligohymenophorea was more abundant in Higashiyachi. Colpodea and Armophorea were prominent in Shiizu, and Prostomatea occurred at relatively high abundance in control treatments at Higashiyachi. Other classes—such as Plagiopylea, Nassophorea, IN 2411, and Karyorelictea—were detected sporadically in individual bottles at some sites but were not consistently observed across replicates. Across sites, more than 53% of ciliophoran OTUs occurred only in experimental treatments, except at Tanakagawa, where the proportion was 37% (Figure 2b). At Usa, which had the lowest total number of ciliophoran OTUs, 96% were unique to experimental treatments. When combined across all sites, 42% of ciliophoran OTUs were exclusive to experimental treatments.

### 3.3 α‐Diversity

**FIGURE 3:**
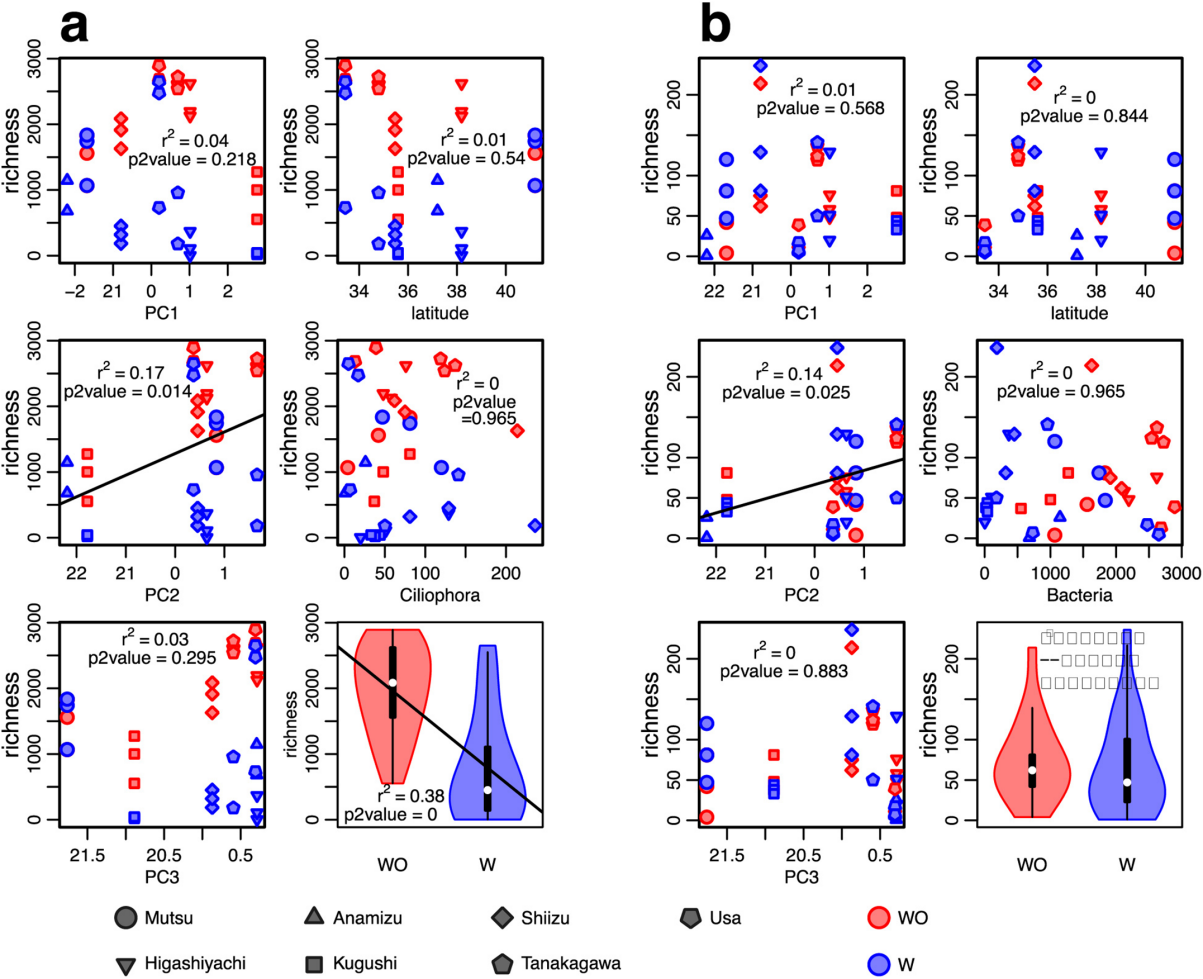
Relationships between OTU‐based α‐diversity and explanatory variables, including principal component (PC) scores, latitude, α‐diversity of the other microbial group, and treatment type. Results are shown separately for (a) bacterial α‐diversity and (b) ciliophoran α‐diversity. Statistically significant regression lines (*p* ≤ 0.05) are shown. Open symbols represent control treatments (WO), and filled symbols represent experimental treatments (W).

In the control treatment without fish carcasses, bacterial α‐diversity was highest at Tanakagawa, with a mean of 2628 OTUs, and lowest at Anamizu, with only 338 OTUs (Figure 3a). Under the experimental treatment with carcasses, Usa exhibited the highest α‐diversity (1952 OTUs), whereas Kugushi showed the lowest (34 OTUs). Overall, bacterial α‐diversity was generally higher in controls than in experimental treatments, except at Mutsu, where values were comparable or slightly higher in the experimental treatment (Figure 3a). Simple regression analysis revealed that bacterial α‐diversity increased significantly with higher PC2 scores, which represent environments with larger tidal ranges and lower temperatures, and was significantly lower in the experimental treatment compared with the control (Figure 3a). No significant relationships were found with latitude, PC1 or PC3 scores, or ciliophoran α‐diversity.

A multiple regression analysis with stepwise selection yielded the following model:

$$\begin{array}{c} \text{Bacterial}\;\alpha -\text{diversity}=-192\times \left[\text{PC1}\right]+269\times \left[\text{PC2}\right]\\ \;-112\times \left[\text{Latitude}\right]-4\times \left[\text{Ciliophoran}\;\alpha -\text{diversity}\right]\\ \;-1167\times \left[\text{Fish carcass}\right]+6307,\;\left(R^{2}=0.608,p<0.001\right). \end{array}$$

This model indicates that bacterial α‐diversity increased under conditions associated with higher PC2 scores but decreased under more oligotrophic conditions (higher PC1 scores), at higher latitudes, with increasing ciliophoran α‐diversity, and in the presence of fish carcasses.

For Ciliophora, α‐diversity in the control treatment was highest at Tanakagawa (127 OTUs) and lowest at Usa (26 OTUs) (Figure 3b). In the experimental treatment, Shiizu exhibited the highest α‐diversity (148.7 OTUs on average), while Usa again showed the lowest (9.7 OTUs). Unlike bacteria, ciliophoran α‐diversity did not differ significantly between treatments (Figure 3b). However, as with bacteria, ciliophoran α‐diversity increased significantly with higher PC2 scores. A multiple regression analysis with stepwise selection identified PC2 as the sole significant predictor, yielding the model:

$$\begin{array}{c} \text{Ciliophoran}\;\alpha -\text{diversity}=17.53\times \left[\text{PC2}\right]+66.56,\\ \;\;\left(R^{2}=0.140,p<0.025\right). \end{array}$$

Thus, unlike bacterial α‐diversity, ciliophoran α‐diversity did not decrease in the presence of fish carcasses.

### 3.4 *β*‐Diversity

**FIGURE 4:**
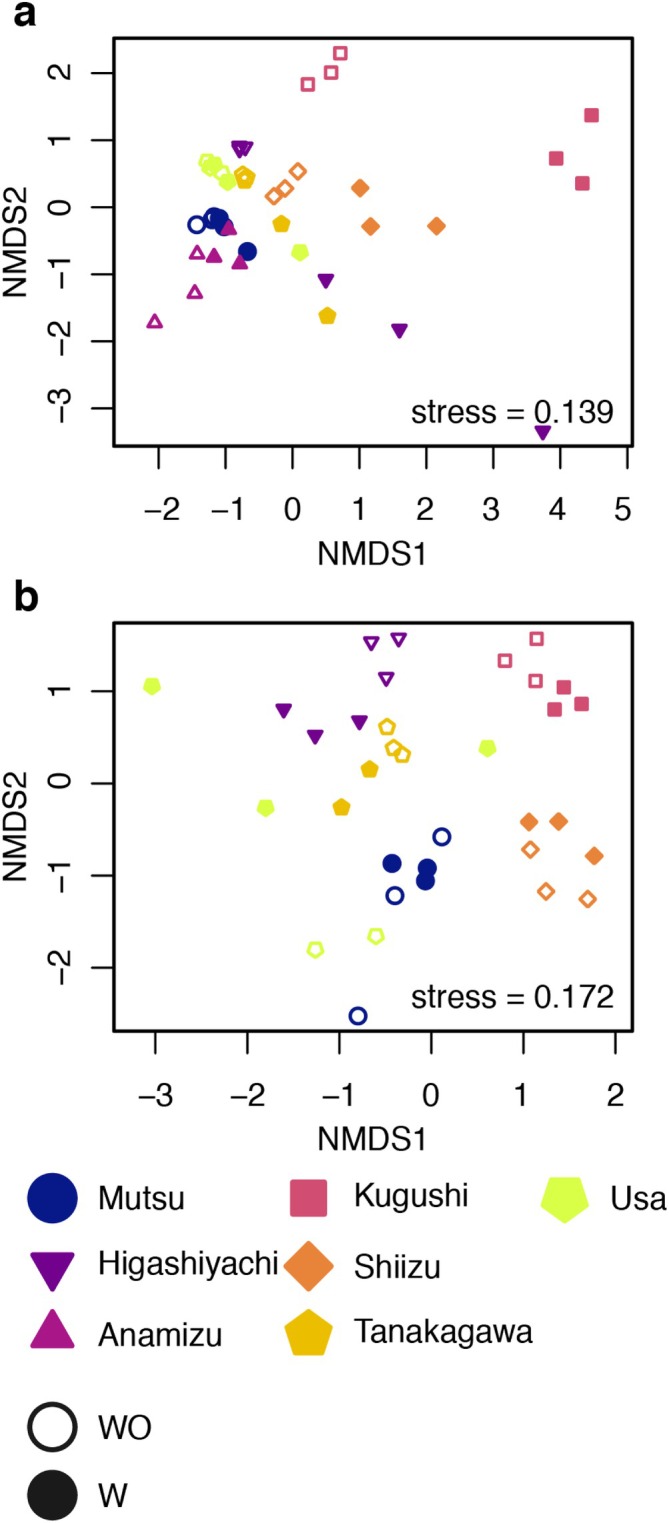
Non‐metric multidimensional scaling (nMDS) biplots illustrating *β*‐diversity of microbial assemblages in control (WO) and experimental (W) treatments at each site. Results are shown separately for (a) bacterial assemblages and (b) ciliophoran assemblages. Different symbols represent sites, and colours represent treatments.

Bacterial *β*‐diversity, calculated using the Horn index, ranged from 0.22 to 1 across sites and treatments. Differences in assemblage structure were visualized by non‐metric multidimensional scaling (nMDS) (Figure 4a). The nMDS plot showed that, with the exception of Mutsu and Usa, bacterial assemblages differed clearly between control and experimental treatments. PERMANOVA confirmed that site (*r* ^*2*^ = 0.489, *p* < 0.001), treatment (*r* ^*2*^ = 0.072, *p* < 0.001), and their interaction (*r* ^*2*^ = 0.194, *p* < 0.001) significantly affected bacterial *β*‐diversity (Table S2).

For ciliophorans, *β*‐diversity ranged from 0.35 to 1. Plots of nMDS revealed that assemblage structures generally clustered by site regardless of treatment, though some treatment‐related divergence was evident (Figure 4b). At Usa, assemblage structures varied widely among replicates, though partial separation by treatment was observed. In contrast, no treatment‐based divergence was detected at Mutsu. As with bacteria, PERMANOVA showed significant effects of site (*r* ^*2*^ = 0.440, *p* < 0.001), treatment (*r* ^*2*^ = 0.045, *p* < 0.001), and their interaction (*r* ^*2*^ = 0.163, *p* < 0.001) on ciliophoran *β*‐diversity (Table S2).

### 3.5 Effects of Fish Carcasses Across Geographically Distinct Sites

**FIGURE 5:**
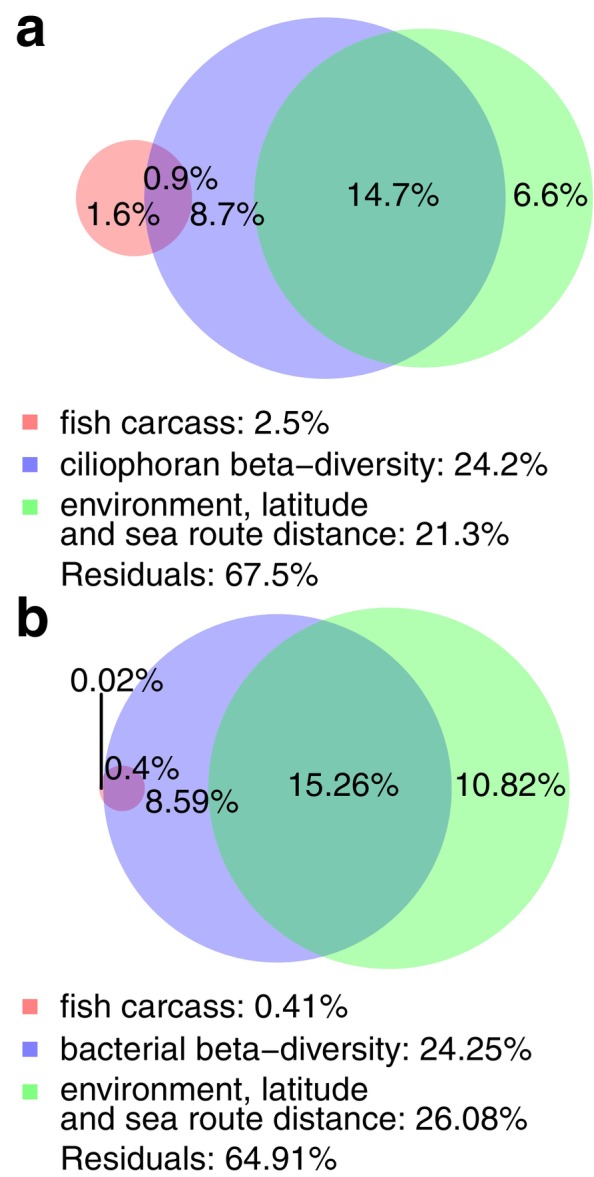
Venn diagrams showing results of variation partitioning analyses explaining *β*‐diversity: (a) bacterial assemblages and (b) ciliophoran assemblages. The area of each section is proportional to the variance explained by each variable, and total coefficients of determination (%) are indicated.

To identify factors influencing bacterial *β*‐diversity across sites and treatments, we performed multiple regression on distance matrices (MRM) (Table S3). Independent variables included Euclidean distances of environmental principal component (PC) scores, latitudinal distance, sea‐route distance, treatment score (0 = same treatment, 1 = different), and pairwise differences in ciliophoran *β*‐diversity. The model explained 33% of the variation in bacterial *β*‐diversity (Figure 5). Variation partitioning showed that ciliophoran *β*‐diversity alone accounted for 8.7% of this variation, and 24.2% when joint effects with other variables were included. In contrast, the fish carcass treatment explained only 1.6% independently and 2.5% when joint effects were considered. Environmental PCs, latitude, and sea‐route distance collectively accounted for 6.6% of the variation, excluding overlaps with other variables.

**FIGURE 6:**
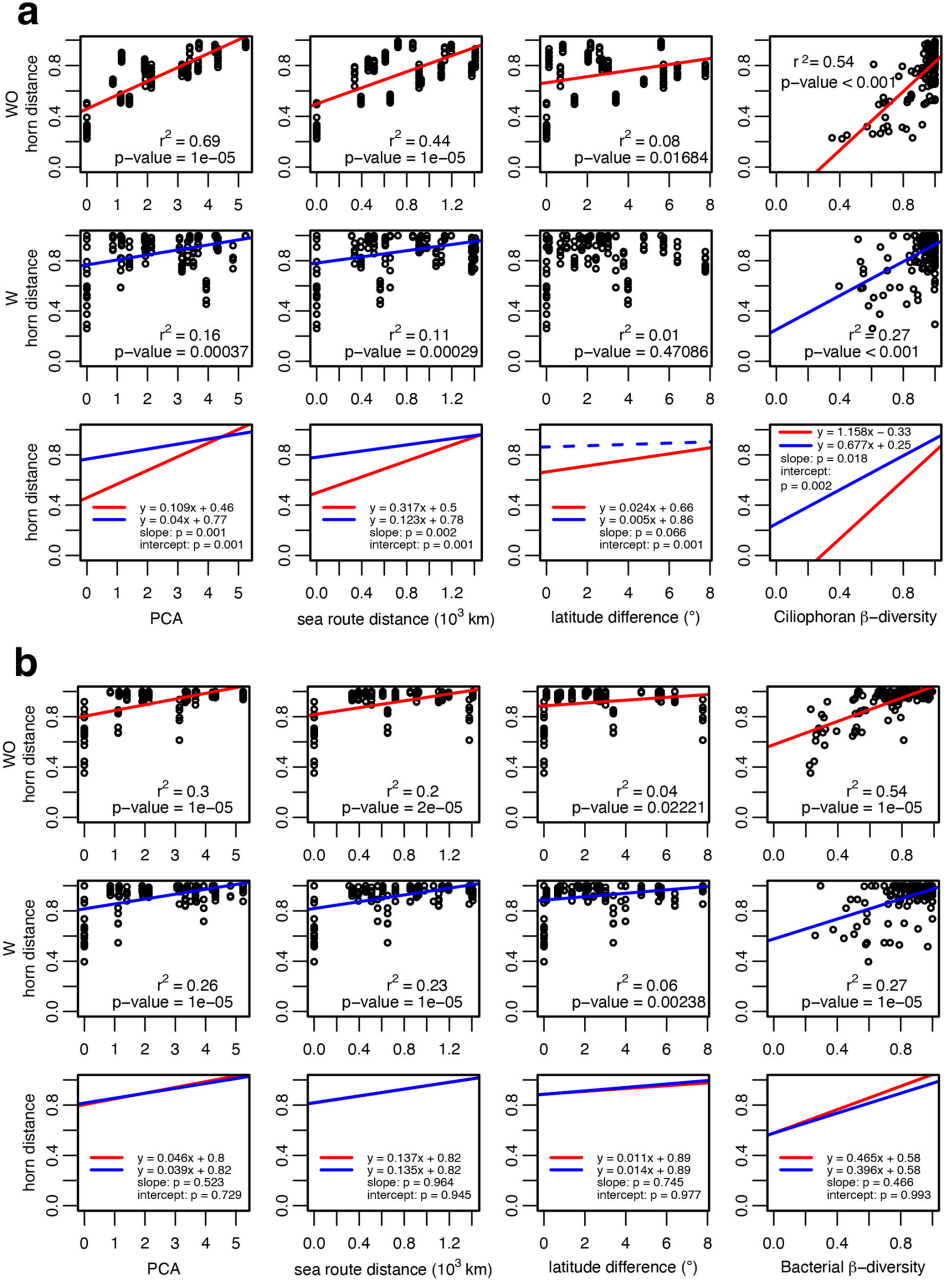
Mantel test results of microbial *β*‐diversity versus environmental and spatial distances for (a) bacteria and (b) ciliophorans. The top row shows control treatments, the middle row shows experimental treatments, and the bottom row displays overlaid regression lines (red = control, blue = experimental). Significant regressions are shown with corresponding R^2^ and *p*‐values, and permutation test results comparing slopes and intercepts are provided.

To test whether these effects differed with carcass presence, Mantel tests were conducted separately for control and experimental treatments (Figure 6a). In both treatments, bacterial *β*‐diversity was positively and significantly correlated with environmental differences (PC scores), ciliophoran *β*‐diversity, and sea‐route distance. However, in the experimental treatment, the Y‐intercept of the regression line was significantly higher (*p* < 0.01) and the slope significantly lower (*p* < 0.05) than in the control, indicating altered sensitivity to these factors in the presence of fish carcasses.

We next assessed how bacterial *β*‐diversity and treatment influenced ciliophoran *β*‐diversity using MRM‐based variation partitioning (Figure 5b). This model explained 35% of the variation. The effect of fish carcass treatment was negligible in explanatory power (0% independently and ≤ 0.4% including joint effects). By contrast, bacterial *β*‐diversity explained 9% independently and up to 24% when joint effects were included.

Mantel tests further showed that ciliophoran *β*‐diversity was significantly correlated with environmental differences, bacterial *β*‐diversity, latitudinal distance, and sea‐route distance in both control and experimental treatments (Figure 6b). Unlike bacteria, no significant differences in regression slope (*p* > 0.05) or Y‐intercept (*p* > 0.05) were detected between treatments, indicating that the factors influencing ciliophoran *β*‐diversity remained consistent regardless of fish carcass presence.

## 4 Discussion

In a previous study, we showed that a fish carcass can induce rapid and substantial shifts in the composition of tidal‐flat microbial communities for 7 days (Kawamoto et al. 2021). However, microbial taxonomic composition is strongly shaped by environmental conditions and geography (Garcia‐Pichel et al. 1999; Wieland and Kühl 2006; Abed et al. 2007; Carrasco and Perissinotto 2012; Zhao and Xu 2017; Li et al. 2019; Jiang et al. 2021; Kawamoto and Urabe 2023). Thus, it remained unclear whether carcass effects on microbial communities would be consistent across tidal flats differing in environmental context. In the present study, we demonstrated experimentally that both bacterial and ciliophoran assemblages in tidal‐flat sediments were significantly altered by the presence of sardine carcasses at all sites across geographically distinct regions of the Japanese archipelago. This finding suggests that animal carcasses generate microbial communities distinct from those in the surrounding sediments, regardless of geographic location.

Across sites, bacterial α‐diversity declined in sediments near sardine carcasses. On average, > 80% of bacterial OTUs observed in the carcass treatment were also present in ambient sediments. These results parallel our earlier study in Sendai Bay, which showed that carcass‐associated bacterial communities were structurally distinct from those in ambient sediments, yet most dominant OTUs originated from the surrounding environment (Kawamoto et al. 2021). A similar trend was observed by Dickson et al. (2011), who found that microbial communities colonizing submerged pig carcasses in seawater were composed mainly of species from the surrounding water. Together, these observations support the idea that microbial colonization near carcasses in aquatic systems is driven primarily by opportunistic taxa from the ambient community.

However, some exceptions were observed. At Kugushi and Anamizu, where OTU richness was low, fewer than half of the OTUs detected in the experimental bottles were shared with the controls. This pattern is similar to that reported by Damann et al. (2015), who found that bacterial taxa dominating human carcasses largely originated from the host gut. If this were also the case in the present study—that is, if bacterial OTUs unique to the experimental treatments originated from sardine carcasses—then bacterial community composition would be expected to be similar between Kugushi and Anamizu. However, this was not observed (Figure 4a). Alternatively, the addition of sardine carcasses may have substantially altered sediment conditions, allowing rare bacterial taxa that were undetectable under ambient conditions to proliferate. This interpretation also suggests that most bacterial OTUs detected in the carcass treatments were derived from the ambient sediment community rather than from the carcasses themselves.

Animal carcasses are ultimately decomposed and assimilated through microbial activity. In the case of sardines placed in tidal flats, carcasses were nearly fully decomposed within 6 weeks, primarily due to microbial processes (Kawamoto et al. 2021). Given the dynamic nature of aquatic environments, ambient bacteria can disperse readily via water movement and rapidly exploit localized, short‐lived habitats such as carcasses, leading to the formation of unique but transient microbial communities.

In contrast to bacteria, ciliophoran α‐diversity was not significantly reduced by the presence of carcasses. Nonetheless, on average > 42% of ciliophoran OTUs in carcass treatments were absent from controls. This finding is consistent with our previous work in Sendai Bay, where a large fraction of ciliophoran OTUs observed near carcasses were absent from ambient sediments (Kawamoto et al. 2021). As mobile protists, ciliophorans may actively migrate toward carcasses in response to increased organic matter or other cues. Kawamoto et al. (2021) also suggest that ciliophorans exhibit greater spatial dispersal capacity than bacteria, which may explain their ability to colonize carcasses even when absent from the local ambient community. It should be noted that, unlike the other sites, the OTU composition of the ciliophoran assemblage showed substantial variation among replicates at Usa (Figure 4b). Because the number of ciliophoran reads was relatively low at this site (Figure 2a), even small differences in the relative abundance of individual OTUs may have been amplified, leading to greater apparent variation in assemblage composition than that observed at the other sites.

Although fish carcasses clearly altered the structure of both bacterial and ciliophoran assemblages, the specific effects varied across sites. For example, Proteobacteria OTUs were relatively more abundant near carcasses at Higashiyachi and Tanakagawa, whereas Firmicutes OTUs increased in carcass treatments at Kugushi and Usa. Consequently, bacterial assemblages near carcasses differed markedly among sites. Similar site‐specific responses were also observed for ciliophoran assemblages. These results support our first hypothesis (H1) that the magnitude of microbial community responses to carcasses depends on site‐specific conditions.

Across sites, OTUs from multiple bacterial phyla were shared between control and carcass treatments. However, Firmicutes and Fusobacteria OTUs were consistently more frequent in bottles containing carcasses. The dominance of Firmicutes during carcass decomposition has also been reported in other contexts, including the decomposition of carp, chicken, and cattle (Yang et al. 2017; Wang et al. 2019; Hilal et al. 2021; Zhou et al. 2021). Fusobacteria are known to thrive under anaerobic conditions. While some taxa are pathogenic and inhabit animal digestive tracts, others—such as Psychrilyobacter—commonly occur in marine sediments and are capable of degrading organic matter, particularly in sulphidic environments (Yadav et al. 2021). The frequent appearance of Firmicutes and Fusobacteria in carcass treatments suggests that decomposition alters ambient conditions, creating localized anaerobic and sulphidic microenvironments in surrounding sediments.

In this study, environmental variables—including temperature, TOC, TN, and particle size—were measured in sediments adjacent to, but not within, the incubation bottles. Nonetheless, 32% of the variation in bacterial *β*‐diversity in tidal‐flat sediments was explained by the variables examined. Fish carcasses significantly affected bacterial *β*‐diversity, but their effect size (2.5%) was much smaller than those of environmental conditions. Similarly, although carcasses significantly influenced ciliophoran *β*‐diversity, the effect size was < 1%. These findings suggest that local environmental conditions exert a much stronger influence on microbial community structure across a large geographic distance than the short‐term impact of carcasses. Previous work showed that microbial assemblages in tidal‐flat sediments were more similar between environmentally similar sites than between geographically proximate ones (Kawamoto and Urabe 2023). Consistent with that finding, our results demonstrate that carcass effects on microbial communities were context dependent and varied among sites with different environmental conditions, even among geographically close tidal flats. This supports our second hypothesis (H2), which posited that microbial assemblages near carcasses would be more similar between environmentally similar sites than between geographically adjacent ones.

Within carcass treatments, bacterial *β*‐diversity increased with greater environmental dissimilarity and geographic distance. However, the slope of this relationship was significantly lower than in control treatments, suggesting that carcasses reduced the expression of site‐specific community characteristics, likely due to nutrient enrichment. High organic input from carcasses may create eutrophic conditions across sites, dampening the influence of local environmental variability on bacterial community structure. For ciliophorans, *β*‐diversity also increased with environmental dissimilarity and geographic distance, but the slope of this relationship did not differ significantly between treatments. This indicates that although carcasses affected ciliophoran taxonomic composition, their overall assemblage structure remained more strongly governed by ambient environmental conditions and spatial factors.

Importantly, 8.7% of the variation in bacterial *β*‐diversity was explained by ciliophoran *β*‐diversity alone, and 14.7% when joint effects were included. A reciprocal pattern was observed for ciliophorans, where bacterial *β*‐diversity explained comparable proportions of variation. These findings suggest that biological interactions—such as predator–prey relationships (Sherr and Sherr 1987; Gonzalez et al. 1990; Epstein 1997) and environmental modifications mediated by either group (Hunter et al. 2006; Chen et al. 2017)—likely contribute to structuring both assemblages. Thus, although our results are correlational, they are consistent with our third hypothesis (H3) that bacterial and ciliophoran assemblages near carcasses may be linked through biological interactions and/or environmental modifications arising from their activities. Previous work on temporal changes in bacterial and ciliophoran assemblages in tidal‐flat sediments suggested that trophic interactions were the primary driver of structural relationships (Kawamoto et al. 2021). However, because the present study did not address temporal dynamics, we cannot fully evaluate the relative importance of trophic versus environmental mechanisms. These mechanisms may also vary among sites depending on local conditions.

## 5 Conclusion

This study demonstrated that animal carcasses significantly alter microbial communities in tidal‐flat sediments, though the magnitude and nature of these effects were strongly site‐specific. However, the influence of carcasses was context dependent, because differences associated with carcass addition were often smaller than the large‐scale spatial variation in microbial community structure among tidal flats across the Japanese archipelago. Importantly, we also found a consistent association between bacterial and ciliophoran assemblages across sites, regardless of carcass presence. This relationship is consistent with the possibility of biological interactions or environmentally mediated processes associated with decomposition, although the underlying mechanisms remain unresolved and require further investigation.

Our analyses focused on community composition rather than function. Although taxonomic shifts in carcass‐associated microbial assemblages were small, these structural changes may nonetheless play an important role in the rates and pathways of fish carcass decomposition. Moreover, the present study involved in situ incubation using only a single fish carcass at each tidal flat. If a larger number of carcasses had been deployed at each site, microbial communities might have become more similar even among distant locations than observed here. Therefore, the present study may underestimate the effects of fish carcasses on local microbial communities. Because animal carcasses are ubiquitous and likely exert important ecological effects on surrounding environments, future studies should integrate structural and functional perspectives. In particular, to evaluate the ecological consequences of carcass inputs in aquatic ecosystems, it is essential to understand how changes in microbial communities are linked to sediment redox conditions, as well as to the rates and processes of nutrient cycling, biogeochemical fluxes, and organic matter degradation.

## Author Contributions

**Yasutake Kawamoto:** conceptualization, investigation, methodology, data curation, formal analysis, visualization, writing – original draft. **Jotaro Urabe:** writing – original draft, writing – review and editing, funding acquisition, project administration, conceptualization, investigation, validation, supervision.

## Funding

This work was supported Japan Society for the Promotion of Science Grant‐in‐Aid for Scientific Research (KAKENHI: 23H02548).

## Conflicts of Interest

The authors declare no conflicts of interest.

## Supporting information


**Figure S1:** Experimental setup. (a) Bottles buried in sediment; (b) bottles covered with a mesh net to prevent displacement; and (c) sediment sampling with a small core sampler inserted through a side hole in the bottle.
**Figure S2:** Mean oxidation–reduction potential (ORP) values in control (WO) and experimental treatments (W) at the end of the experiment across sites. Each treatment was replicated three times per site.
**Figure S3:** Relative abundances of microbial assemblages based on high‐throughput sequencing: (a) bacterial phyla from 16S rRNA sequencing and (b) ciliophoran classes from 18S rRNA sequencing. Comparisons are shown between control (WO) and experimental (W) treatments.
**Table S1:** Sea‐route distances (km) between the study sites.
**Table S2:** Results of PERMANOVA to examine the effects of the fish carcasses and sites on bacterial and ciliophoran assemblages.
**Table S3:** Results of multiple regression on distance matrices (MRM) identifying factors influencing β‐diversity of bacterial and ciliophoran assemblages across sites and treatments. Model and predictor significance were assessed using 10,000 permutations. Reduced models (e.g., non‐full models) were used for variation partitioning of each variable.

## Data Availability

The data of the bacterial and ciliophoran assemblage metagenome were deposited in the DNA Data Bank of Japan Sequence Read Archive under the accession numbers from DRR453415 to DRR453498 (https://www.ddbj.nig.ac.jp/dra/index.html). Sequences from the negative control treatments were also deposited under the accession numbers DRR453402, DRR453403, DRR453413, and DRR453414.
