# Correction to ‘Geographic Variability of Fish Carcass–Associated Microbial Communities’

**DOI:** 10.1111/1758-2229.70407

**Published:** 2026-08-26

Kawamoto, Y. , and J. Urabe . 2026. “Geographic Variability of Fish Carcass–Associated Microbial Communities.” Environmental Microbiology Reports 18, no. 4: e70389. 10.1111/1758-2229.70389.42487460 PMC13392499

In Figure 3, the term ‘p2value’ in each panel is a typographical error. The correct term is ‘*p*‐value.’ Additionally, the statistics in the bottom‐right panel is missing and should read ‘*R* ^2^ = 0.01, *p*‐value = 0.68’. The revised figure is shown below:

**FIGURE 3:**
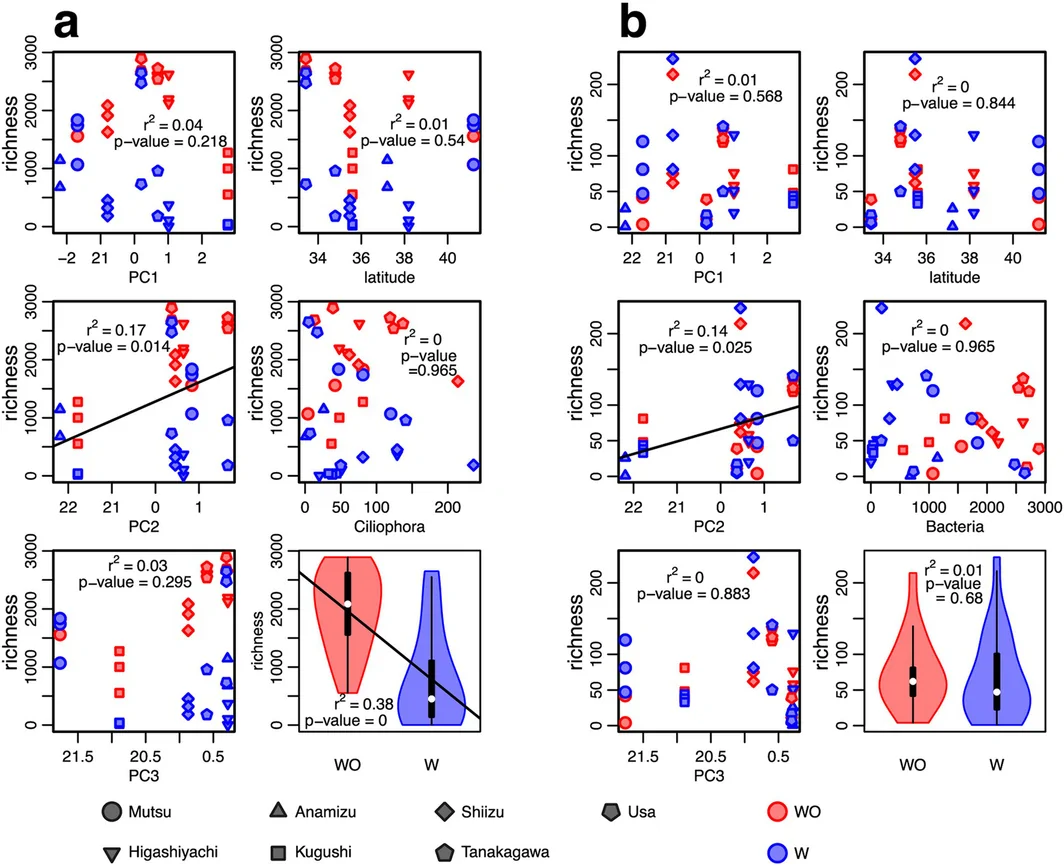
Relationships between OTU‐based α‐diversity and explanatory variables, including principal component (PC) scores, latitude, α‐diversity of the other microbial group, and treatment type. Results are shown separately for (a) bacterial α‐diversity and (b) ciliophoran α‐diversity. Statistically significant regression lines (*p* ≤ 0.05) are shown. Open symbols represent control treatments (WO), and filled symbols represent experimental treatments (W).

These changes do not alter the conclusions of the article.

We apologize for this error.

