# Supplementary material for: Geographic Variability of Fish Carcass–Associated Microbial Communities
Source: Environ Microbiol Rep. 2026 Jul 22;18(4):e70389. doi: 10.1111/1758-2229.70389 (PMC13392499; doi:10.1111/1758-2229.70389)
Supplement: Supplementary file 1 — Figure S1: Experimental setup. (a) Bottles buried in sediment; (b) bottles covered with a mesh net to prevent displacement; and (c) sediment sampling with a small core sampler inserted through a side hole in the bottle. Figure S2: Mean oxidation–reduction potential (ORP) values in control (WO) and experimental treatments (W) at the end of the experiment across sites. Each treatment was replicated three times per site. Figure S3: Relative abundances of microbial assemblages based on high‐throughput sequencing: (a) bacterial phyla from 16S rRNA sequencing and (b) ciliophoran classes from 18S rRNA sequencing. Comparisons are shown between control (WO) and experimental (W) treatments. Table S1: Sea‐route distances (km) between the study sites. Table S2: Results of PERMANOVA to examine the effects of the fish carcasses and sites on bacterial and ciliophoran assemblages. Table S3: Results of multiple regression on distance matrices (MRM) identifying factors influencing β‐diversity of bacterial and ciliophoran assemblages across sites and treatments. Model and predictor significance were assessed using 10,000 permutations. Reduced models (e.g., non‐full models) were used for variation partitioning of each variable. [file EMI4-18-e70389-s001.pdf]

Supporting information

GEOGRAPHIC VARIABILITY OF FISH CARCASS–ASSOCIATED MICROBIAL COMMUNITIES

Yasutake Kawamoto and Jotaro Urabe

Figure S1.

Figure S2.

Figure S3.

Table S1

Table S2

Table S3

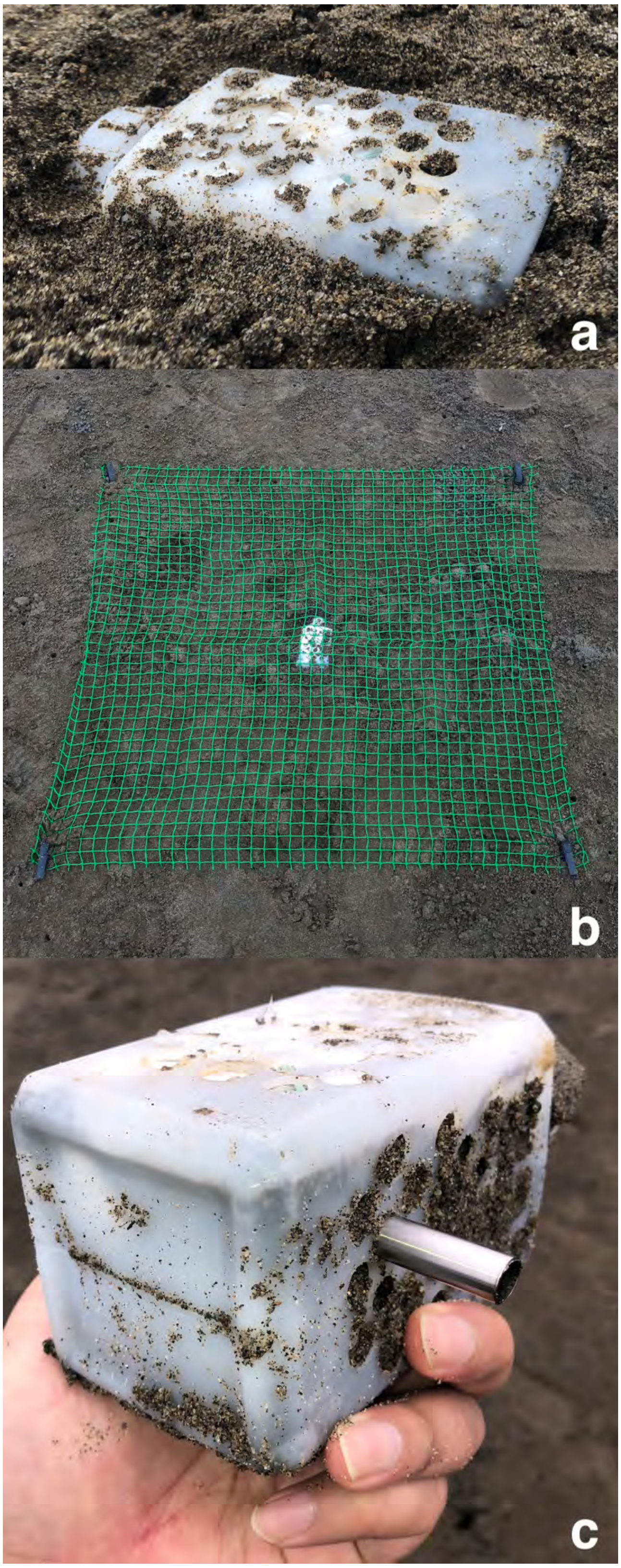

Figure S1. Experimental setup. (a) Bottles buried in sediment; (b) bottles covered with a mesh net to prevent displacement; and (c) sediment sampling with a small core sampler inserted through a side hole in the bottle.

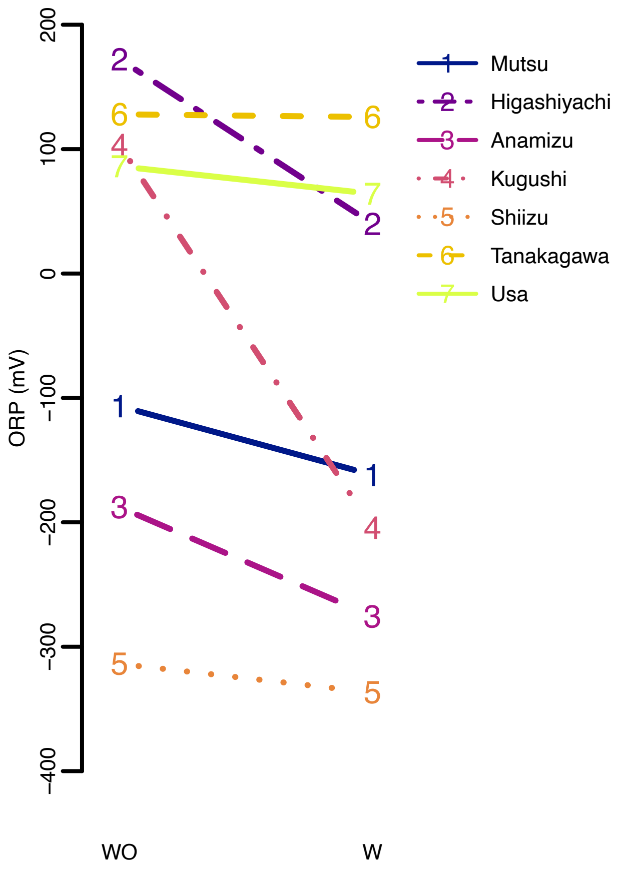

Figure S2. Mean oxidation–reduction potential (ORP) values in control (WO) and experimental treatments (W) at the end of the experiment across sites. Each treatment was replicated three times per site.

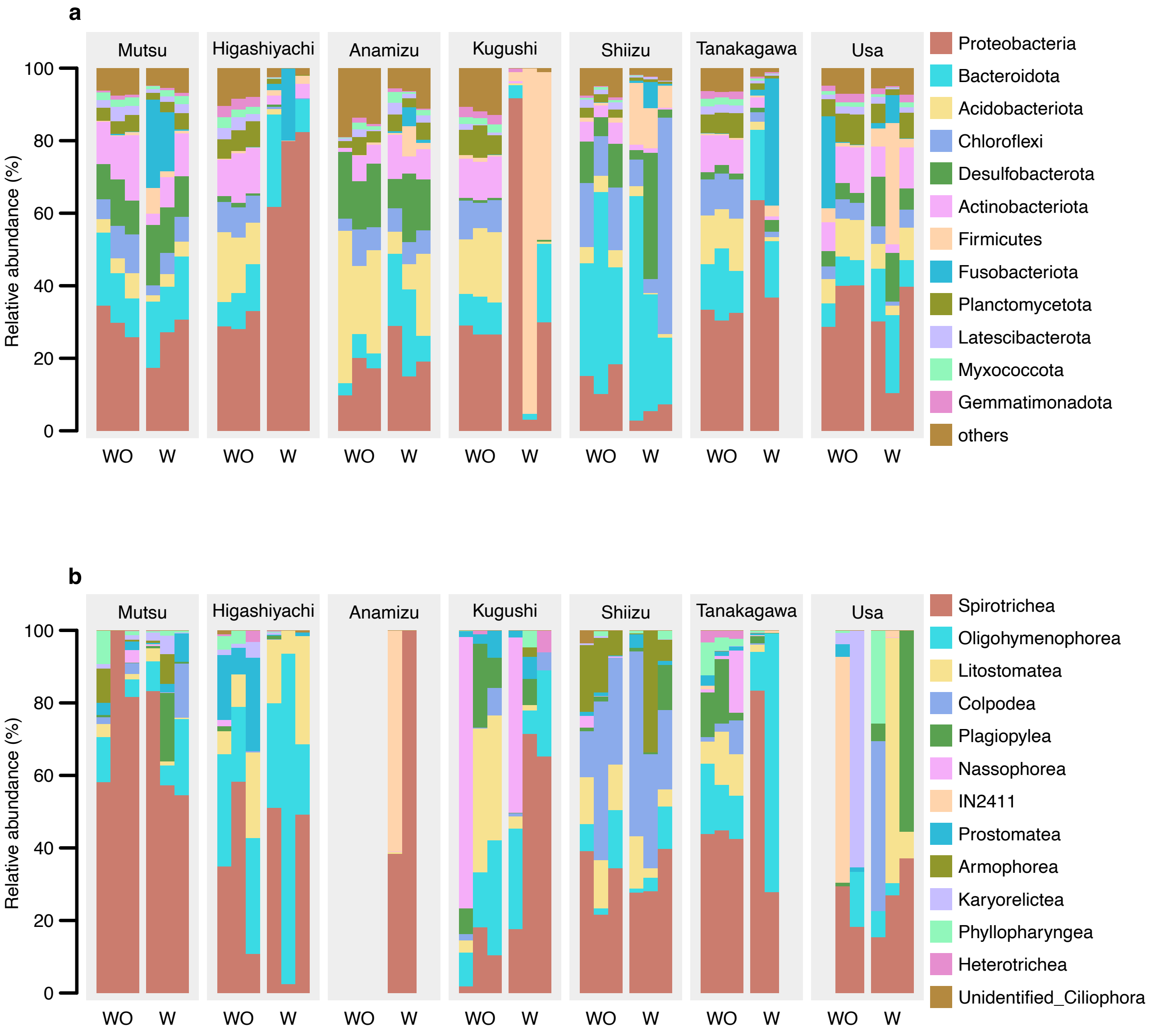

Figure S3. Relative abundances of microbial assemblages based on high-throughput sequencing: (a) bacterial phyla from 16S rRNA sequencing and (b) ciliophoran classes from 18S rRNA sequencing. Comparisons are shown between control (WO) and experimental (W) treatments.

Table S1. Sea-route distances (km) between the study sites.

(km)	Kawauchi	Sendai	Anamizu	Tsuruga	Anesaki	Chiyozaki
Sendai	510					
Anamizu	563	978				
Tsuruga	723	1138	311			
Anesaki	909	457	1378	1410		
Chiyozaki	1106	653	1402	1194	336	
Usa	1379	927	1061	853	610	394

Table S2. Results of PERMANOVA to examine the effects of the fish carcasses and sites on bacterial and ciliophoran assemblages.

Bacteria	d.f.	SS	MS	F-value	r^2	P
Site	6	7.287	1.215	8.966	0.489	<0.001
Fish	1	1.068	1.068	7.888	0.072	<0.001
Site × Fish	6	2.897	0.483	3.564	0.194	<0.001
Residuals	27	3.657	0.135		0.245	
Total	40	14.910				
**Ciliophorans**	**d.f.**	**SS**	**MS**	**F-value**	r^2	**P**
Site	5	6.310	1.262	5.518	0.440	<0.001
Fish	1	0.649	0.649	2.839	0.045	<0.001
Site × Fish	5	2.342	0.468	2.048	0.163	<0.001
Residuals	22	5.031	0.229		0.351	
Total	33	14.332				

Table S3. Results of multiple regression on distance matrices (MRM) identifying factors influencing β-diversity of bacterial and ciliophoran assemblages across sites and treatments. Model and predictor significance were assessed using 10,000 permutations. Reduced models (e.g., non-full models) were used for variation partitioning of each variable.

Model	treatment score		ciliophoran β-diversity		environmental PC		latitudinal distance		sea route distance		y-intercept		r^2	
Full model	0.0525	***	0.5501	***	0.0310	**	-0.0068		0.0258		0.2229	***	0.3299	***
without environmental PC, latitudinal distance and sea route distance	0.0456	***	0.7774	***	-		-		-		0.0965	***	0.2585	***
without treatment score	-		0.5776	***	0.0300	**	-0.0071		0.0232		0.2295	***	0.3088	***
without ciliophoran β-diversity	0.0632	***	-	-	0.0440	***	-0.0057		0.0559	*	0.6692	***	0.2441	***
only treatment score	0.0568	***	-	-	-		-		-		0.8132	***	0.0250	***
only ciliophoran β-diversity	-		0.7906	***	-		-		-		0.1076	***	0.2425	***
only environmental PC, latitudinal distance and sea route distance	-		-	-	0.0436	***	-0.0059		0.0545	*	0.7044	**	0.2131	***

Model	treatment score		bacterial β-diversity (upper		environmental PC		latitudinal distance		sea route distance		y-intercept		r^2	
Full model	0.0064		0.2065	***	0.0145	***	0.0032		0.0431		0.6731	***	0.3514	***
without environmental PC, latitudinal distance and sea route distance	-0.0031		0.3080	***	-		-		-		0.6714	***	0.2426	***
without treatment score	-		0.2105	***	0.0143	***	0.0032		0.0427	***	0.6739	***	0.3507	***
without bacterial β-diversity	0.0194	*			0.0236	***	0.0020		0.0546	***	0.8114		0.2683	***
only treatment score	0.0144				-		-		-		0.9219		0.0041	*
only bacterial β-diversity	-		0.3067	***	-		-		-		0.6710	***	0.2425	***
only environmental PC, latitudinal distance and sea route distance	-		-		0.0235	***	0.0020		0.0542	***	0.8222		0.2608	***

Signiant level: *** p<0.001; ** p<0.01; and * p<0.05
